# Serum alpha–actinin antibody status in systemic lupus erythematosus and its potential in the diagnosis of lupus nephritis

**Published:** 2016

**Authors:** Mansour Babaei, Zahra Rezaieyazdi, Nayereh Saadati, Massoud Saghafi, Maryam Sahebari, Bahram Naghibzadeh, Habibollah Esmaily

**Affiliations:** 1 Department of Rheumatology, Ayatollah Rouhani Hospital, Babol University of Medical Sciences, Babol, Iran.; 2 Rheumatic Disease Research Center, School of Medicine, Mashhad University of Medical Sciences, Mashhad, Iran.; 3 Center for Health Sciences Research, School of Health, Mashhad University of Medical Sciences, Mashhad, Iran.

**Keywords:** Systemic lupus erythematous, Nephritis, Anti-alpha-actinicn antibody, Diagnosis.

## Abstract

**Background:**

In lupus nephritis (LN), deposition of pathogenic autoantibodies in the glomeruli is mediated via cross-reactivity with alpha-actinin. Association of serum alpha-actinin antibody (AαA) with LN has been shown in a few studies but the results are controversial.

**Methods:**

Eighty patients into entered the study. The diagnosis of SLE was confirmed according to the American College of Rheumatology criteria and LN was diagnosed by proteinuria ≥ 500 mg/24 hour and kidney biopsy. Serum AαA was measured with ELISA method. Receiver operating characteristics curve (ROC) analysis was applied to determine an optimal cutoff value for AαA to discriminate patients with and without LN at the highest sensitivity and specificity. The association of AαA with LN was determined by logistic regression analysis with calculation of odds ratio (OR).

**Results:**

Serum AαA was significantly lower in LN as compared with SLE patients without LN (P=0.001). Serum AαA at cutoff levels ≤ 59.5 pg/ml discriminated the two groups with sensitivity, specificity, positive predictive values of 60%. 90% and 85.7%, respectively. Serum AαA level ≤ 59.5 pg/ml was significantly associated with LN (OR=13.5, P=0.001) and the OR increased to 25.2 (P=0.003) after adjustment for age, sex, C3, C4, anti-ds-DNA, SLEDAI.

**Conclusion:**

This study indicates that serum AαA decreases in LN and serum level ≤ 59.5 pg/ml is SLE and is predictive of nephritis

Systemic lupus erythematous (SLE) is associated with multiple organ involvement and high morbidity as well as mortality (1-4). Among the several manifestations of SLE, nephritis is of particular concern ,because lupus nephritis (LN) is associated with excess risk of death, malignancy and cardiovascular complications (3, 4). Alpha-actinin (α-actinin) is a ubiquitous cytoskeletal protein which belongs to the superfamily of filamentous actin (F-actin) crosslinking proteins. It is present in multiple subcellular regions of both muscle and non-muscle cells, including cell–cell and cell–matrix contact sites, cellular protrusions and stress fiber dense regions. It seems to bear multiple important roles in the cell by linking cytoskeleton to many different transmembrane proteins in a variety of junctions. Deposition of autoantibodies in the glomeruli seems to be crucial for development of LN (5). 

In SLE, anti – alpha-actinin (AαA) is a major cross-reactive target for anti-dsDNA antibodies (6) and pathogenicity of some anti-DNA antibodies is mediated via cross-reactivity with alpha-actinin (7-9). Earlier studies have shown that renal pathogenicity of murine lupus antibodies are dependent on direct binding of antibodies to AαA (7-9). Active LN compared with SLE patients without nephritis displays greater AαA binding (6). It has been shown that pathogenic anti-ds DNA antibodies bind strongly to AαA and elevated levels of serum AαA antibodies are associated with a 2.5 -fold increase in the prevalence of nephritis (6). In one study, 10 out of 22 (45.1%) patients with AαA antibody had LN, while only 14 out of 78 (17.9%) SLE patients without AαA antibody had LN. This indicates a significant association between serum AαA antibody and LN (6). Nevertheless, SLE patients without nephritis and even patients without SLE may also have serum AαA antibody (8-10). Croqueted et al. compared the prevalence of AαA, between SLE and other rheumatic diseases versus healthy controls (9). 

The results showed higher prevalence of AαA antibody in SLE compared with rheumatoid arthritis, Siogren syndrome, and healthy controls (22.3%, 3.92%, 3%, and 0.6% respectively). In Renaudineau et al,’s study (6), the prevalence rate of AαA antibody positivity was higher in anti-dsDNA positive versus anti-dsDNA negative SLE (33.8% vs 2.8%). Nevertheless, in a longitudinal study of 16 patients with new-onset-biopsy-proven LN, there was a positive association between measures of LN with anti-DNA and anti-nucleosome but not with AαA antibody (11).

In a few studies, the relationship between serum AαA antibody and SLE disease activity index (SLDAI) or anti-ds DNA was assessed and the results revealed a negative correlation of AαA with SLEDA and positive correlation with anti-dsDNA (6, 9, 10, 12).

 Available data indicate that binding of pathogenic autoantibodies to AαA antibody is critical for the development of nephritis in SLE, suggesting a relationship between serum AαA antibody and LN. Nevertheless, the results of studies in this context are controversial (6, 10, 11, 12) which may be attributed to inadequate sample size, inappropriate study designs, patient selection or nonhomogeneous distribution of predisposing factors of LN across various studies. To overcome these shortcomings, the present case- control study was designed to compare SLE patients with and without nephritis regarding serum AαA antibody levels and to investigate the relationship between serum AαA antibody and LN. The secondary purpose of this study was to determine a cutoff level of AαA antibody for the discrimination of SLE patients with and without nephritis. 

## Methods

Ninety patients with lupus erythematous were recruited consecutively according to inclusion criteria among those who presented to rheumatology clinics of Mashhad University of Medical Sciences, Mashhad, Iran, Patient selection was performed over one year period from October 2011 to September 2012. 

The diagnosis of SLE was confirmed by the American College of Rheumatology criteria for systemic lupus erythematous (13) and the diagnosis of LN was confirmed in the presence of >  500 mg per 24 hours proteinuria for at least two occasions as well as kidney biopsy (13). The activity of SLE (SLEDAI) was assessed by a validated questionnaire for SLE disease activity (14). All patients with confirmed LN were included. 

Exclusion criteria included SLE patients with diabetes, urinary tract infection or urinary nephrolithiasis, patients with overlap connective tissue disease, vasculitis syndrome, SLE patients with antiphospholipid syndrome, end- stage renal disease or patients on hemodialysis. SLE patients without proteinuria were considered as controls.

Sample size was estimated for detection of 30 % differences in proportion of AαA antibody positivity between SLE patients with and without nephritis. Based on an earlier prevalence of 15% AαA antibody positivity in SLE patients without nephritis, (6) a sample size of 33 patients for each group was needed to detect such difference with 95% confidence interval (CI) and 80% power (15). However, we recruited additional patients to compensate the patients with missed data. All patients received appropriate treatment for SLE or LN to achieve remission. This study was confirmed by the Ethics Committee of the Mashhad University of Medical Sciences, Mashhad, Iran. Serum AαA antibody level was assessed with ELISA method according to manufacturer’s instruction using human alpha-actinin-4 kit (ACTN-4) ELISA kit CSB -E147 42h (96T) purchased from CUSABIO company. 

**Statistical analysis:** Receiver operating characteristics ROC curve analysis was applied by plotting sensitivity against 1-specificity for various levels of serum AαA antibody .The optimal cutoff value that best distinguished patients with LN from those without LN was determined at maximum value for Youdens' index defined as the difference between the true positive rate and the false positive rate [sensivitiy - (1-specificity)]. The overall diagnostic accuracy was estimated based on area under the ROC curve (AUC) expressed as mean ± SE. 

 In additional analysis the levels of serum AαA antibody in patients with and without LN were compared with other predictive measures of nephritis like C3, C4, anti-ds DNA, serum creatinine (Cr). The status of distribution for all variables was examined by measures of skewness and kurtosis as well as using Kolmogrov -Smirnov test. Normality of distribution was assessed by Kolmogrov-Smirnov test. Parametric tests were used for the comparison of variables with normal distributions and nonparametric Mann-Whitney U test for comparison of skewed variables. Proportions were compared with chi -square or Fisher’s exact tests as appropriate. Association among categorical variables was determined by chi-square test with calculation of odds ratio (OR) and corresponding 95% confidence interval (95%CI). Correlations were performed using Spearman correlation coefficient.

## Results

Eighty participants (95% females) achieved inclusion criteria that comprised 40 patients with LN with mean age of 29.9±19.7 years old and 40 controls without LN with mean age of 30.7±10.7 years old (P=0.63) (table 1). In the total number of patients, 54 (67.5%) patients were anti-ds DNA positive, 46 (57.5%) had low C3 levels, and 37 (46.3%) with low serum C4 levels. Distribution of serum AαA antibody in the control group (patients without LN) was normal with mean value of 124±56.2 pg/ml and median value of 121 pg/ml ,but in patients with LN , distribution of serum AαA antibody was skewed to the right with mean value of 78.2±56.9 pg.ml and median value of 50 pg/ml (P=0.001). 

Based on the results of ROC curve analysis, serum AαA antibody level of ≤ 59.5 pg/ml yielded the highest Youden’s index value for discriminating patients with and without LN at sensitivity of 69% and specificity of 90%. At this level, serum AαA antibody exhibited a false positive rate of 10% and positive predictive value of 85.7% (95% confidence interval 66.4-95.3) and prevalence weighted likelihood ratio of 6 for diagnosis of LN (95% CI, 2.39-15).

 Serum AαA antibody at cutoff level of ≤ 59.5 pg/ml exhibited an AUC (±SE) value of 0.701±0.065 indicating 70.1%. The results of ROC curve analysis regarding other measures of SLE did not show significant ability in predicting LN (table 2). Association of AαA antibody with LN serum AαA antibody ≤ 59.5 pg/ml was significant by OR= 13.5 (95% CI, 4.05-45.3, P=0.001. After adjustment for age, sex, anti-ds DNA, C3,C4, creatinine, SLEDAI, serum AαA ≤ 59.5 pg, ml was independently associated with LN by adjusted odds ratio of 25.2 (95% CI, 3.02- 211.4, P=0.003). While the association of LN with anti-dsDNA, age, sex, C4, and creatinine did not reach to a statistically significant level. But serum C3 levels ≤ 12.5 U/ml were significantly associated with LN by adjusted odds ratio = 8.96 (95% CI, 1.114- 70.3, P=0.037).

**Table 1:** Characteristics of systemic lupus erythematous patients with and without lupus nephritis

**Variable**	**Control (LN-)**	**Patients (LN +)**	**P value** ¥
Age, years mean±SD	30.7±10.7	29.9±9.6	0.720
anti-*α*-actinin Abs (pg/ml)	124.2±56.2	78.2±56.9	0.001
SLDAI-2k ^V^	11.4±13.0	17.6±11. 1	0.057
Anti-ds-DNA Positivity N(%)	25 (46.3%)	29 (53.7%)	0.23
C3( mg/dl)	68.6±42.7	59±45.4	0.353
C4 (mg/dl)	21.5±11.1	20.2±14.9	0.656
ESR (mm/h)^€^	85.47±54.8	104.0±58.1	0.234
Serum creatinine (mg/dl)	0.2±0.2	0.8±0.7	0.115

P value < 0.05 is significant.

¥ Compared by Mann-Whitney U test ≠ Anti-alpha-actinin antibody

҂ Systemic lupus erythematosus disease activity index

€ Erythrocyte sedimentation rate

Anti-ds DNA and LN: Twenty- nine patients with LN (53.7%) versus 25 controls without LN (46.3%) were anti-ds DNA positive (p=0.23). The levels of AαA antibody did not differ between the DNA negative and DNA positive groups (110±59.5 vs 96.8±61.4 pg/ml, p=0.36). In patients with LN serum AαA antibody was negatively correlated with SLEDAI (Spearman’s correlation coefficient= -0.352, P=0.05) but positively correlated with serum C3 level (r=0.419, P=0.014) as well as serum C4 level (r=0.335, P=0.05). Inasmuch as in the control group correlation between AαA antibody and SLEDA, C3 and C4 did not reach to statistically significant levels.

**Table 2:** Diagnostic performance of AαA antibody in the differentiation of SLE patients with and without nephritis in comparison to other conventional markers of lupus nephritis

**Variable**	**Cutoff value**	**Sensitivity**	**Specificity**	**AUC ± SE (95%CI) ** *	**P-value**
Anti-α-actinin Antibody(pg/ml)	59.5	60	90	0.701 ± 0.0 (0.580-0.834)	0.002
C3 U/ml	29.5	38.2	90	0.603±0.068 (0.469--.737)	0.12
C4 U/m	12.5	48.5	71	0.573±0.07 (0.437=0.710)	0.28
Creatinine mg/dl	0.95	56.6	27.5	0.607±0.065 (0.470-0.735)	0.1

* Using receiver operating characteristics curve (ROC) analysis

## Discussion

The findings of this study indicate significantly lower serum AαA antibody concentration in SLE with nephritis as compared to those without nephritis. In LN, low levels of serum AαA antibody correlated positively with serum C3, C4 and creatinine but negatively correlated with SLEDAI. The levels of AαA antibody levels ≤ 59.5 pg/ml distinguished SLE with and without nephritis with sensitivity of 60%, specificity of 90%, positive predictive value of 85.7 % with likelihood ratio of 6. In addition, serum AαA antibody ≤ 59.5 pg/ml was significantly associated with LN after adjustment for other associated risk factors such as anti-ds DNA positivity, low serum complement levels, sex, age, SLE activity by adjusted OR of 25.2.

In this study, serum AαA antibody was not associated with anti-dsDNA which is in contrast with the results of Renaudineau et al. who have found a positive association between LN and anti-ds-DNA (6). Nonetheless, the association was only limited to anti-dsDNA positive nephritis (6). Similarly, Croquefer et al. found higher prevalence rate of AαA antibody positivity in SLE as compared with other rheumatic diseases as well as healthy controls regardless of nephritis (9). Similar to our study, Zhang et al, also reported an inverse relationship between serum AαA antibody and disease activity in SLE irrespective of LN (10). In another longitudinal study of 16 patients with LN, Manson et al. found higher baseline anti-dsDNA and anti-nucleosome but not AαA antibody in SLE than in the healthy controls. In the latter study, serum AαA antibody had not been compared between patients with and without nephritis and the authors found no association between serum AαA antibody and associated factors of nephritis (16). In another case-control study by Becker- Merok et al. (12), serum AαA antibody was higher in anti-dsDNA positive SLE than other autoimmune rheumatic diseases and the serum AαA antibody was higher in renal flare and was independently correlated with anti-dsDNA. Notwithstanding, the association in this study was not SLE specifically because serum AαA antibody was not higher in other ANA positive autoimmune disease. Therefore, the observed association of serum AαA antibody and renal disease suggests cross-reactivity of AαA antibody with anti-dsDNA antibodies (12).

Cross-reactivity of anti-dsDNA and AαA antibody has been shown in a panel of 10 anti-dsDNA and/or AαA antibodies generated by Epstein Barr virus transformation of lymphocytes from patients with SLE. The results provided strong support for contribution of pathogenic cross-reactive anti-dsDNA/ AαA antibody in the development of LN (17). In spite of many previously published studies regarding serum AαA antibody in SLE, yet the status of the serum AαA antibody in SLE patients with and without nephritis has not been addressed. The results of this study in consistent with similar reports (10, 18) present additional information to the existing data concerning the ability of this antibody in recognizing LN. LN is one of the most serious manifestations of SLE and a predictor of morbidity and mortality in these patients (3, 19). Early diagnosis and treatment of LN is of particular importance because treatment at earlier stage, prevents intractable kidney disease. Currently, the diagnosis of LN is based on clinical or laboratory findings which do not always correlate with pathologic abnormalities and thus, the diagnosis warrants certainty (20).

Although, renal biopsy is the gold standard method of diagnosis but it is an invasive procedure and the results of biopsy do not always provide additional benefits compared with clinical classification (21). Based on the findings of this study, serum AαA antibody ≤ 59.5 pg/ml provides supporting data in diagnosing LN with sensitivity of 60% and specificity of 90%. Diagnostic rate of LN in the clinical setting of the present study increased from the pre-test probability of 50% to post- test probability of 85.7%. Excellent likelihood ratio in this study indicates that the post-test probability is less subjected to sample bias. 

Concerning the 50% prevalence of nephritis across various studies (22024), the population of this study should be considered the representative of SLE in general population. Several biomarkers were used for the diagnosis of nephritis in SLE, but none of them was validated in prospective studies and their performance may differ in various ethnic backgrounds (25, 26, 27).

The findings of this study should be considered with limitation since a number of SLE with asymptomatic LN may be missed because of lack of biopsy. Hence, the real number of LN may be underestimated. The strength of this study depends on the study population which was drawn from a homogenous population concerning ethnic and sociodemographic characteristics, treatment as well as diagnostic criteria. Another strength of this study is related to the study design consisted of two groups of SLE patients with similarity in many baseline characteristics including age, sex, renal function, serum complement levels and proportion of anti-dsDNA positivity. Adequate sample size and application of ROC curve analysis provides additional documents for validity. In conclusion, the findings of this study indicate that serum AαA antibody level is significantly higher in SLE with nephritis and at serum cutoff level ≤ 59.5 pg/ml differentiates SLE patients with and without nephritis with sensitivity of 60%, specificity of 90%. Serum AαA ≤ 59.5 ≤ 59.5 pg/ml is significantly associated with LN and yields a positive predictive value by 85.7%. The findings of this study require to be confirmed by longitudinal studies with biopsy-proven LN.

## Notes

**Funding:** The Research Council of Mashhad University of Medical Sciences, financially supported this project with grant number 900486.
